# Association of dietary patterns with blood uric acid concentration and hyperuricemia in northern Chinese adults

**DOI:** 10.1186/s12937-022-00789-7

**Published:** 2022-06-23

**Authors:** Meiqi Zhou, Xin Huang, Ruiqiang Li, Zechen Zhang, Limin Zhang, Xian Gao, Hui Yang, Yuxia Ma

**Affiliations:** 1 Department of Nutrition and Food Hygiene, School of Public Health, Hebei Province Key Laboratory of Environment and Human Health, Hebei Medical University, 361 Zhongshan East Road, Hebei Province 050017, Shijiazhuang, China; 2 Handan Center for Disease Control and Prevention, Handan, China; 3 Experimental Center, School of Public Health, Hebei Province Key Laboratory of Environment and Human Health, Hebei Medical University, 361 Zhongshan East Road, Hebei Province 050017, Shijiazhuang, China

**Keywords:** Dietary pattern, Hyperuricemia, Principal component analysis, Reduced-rank regression, Partial least-squares

## Abstract

**Background:**

Studies have shown that a direct association exists between the diet and blood uric acid concentrations. However, works on the association of dietary patterns with blood uric acid concentrations and hyperuricemia remain limited.

**Objective:**

This study aims to evaluate the association of dietary patterns with blood uric acid concentrations and hyperuricemia.

**Methods:**

The relationship between dietary patterns and hyperuricemia was explored through a nutritional epidemiological survey in China (*n* = 4855). Three statistical methods, including principal component analysis, reduced rank regression (RRR), and partial least squares regression, were used to extract dietary patterns. General linear regression and logistic regression analyses were utilized to explore the relationship of dietary patterns with blood uric acid concentrations and hyperuricemia.

**Results:**

After adjusting for potential confounding factors, the score for the plant-based dietary pattern was found to be negatively correlated with blood uric acid levels (β =  − 3.225) and that for the animal dietary pattern was discovered to be directly correlated with blood uric acid levels (β = 3.645). The participants in the highest quartile of plant-based dietary pattern scores were at a low risk of hyperuricemia (OR = 0.699; 95% CI: 0.561–0.870, *P* < 0.05), whereas those in the highest quartile of animal dietary pattern scores were at a high risk of hyperuricemia (OR = 1.401; 95% CI: 1.129–1.739, *P* < 0.05). The participants in the third quartile of scores for the RRR dietary pattern, which was characterized by the relatively high intake of poultry, sugary beverages, and animal organs and the low intake of desserts and snacks, had a significantly higher risk of hyperuricemia than those in the first quartile of scores for the RRR dietary pattern (OR = 1.421; 95% CI: 1.146–1.763, *P* < 0.05).

**Conclusions:**

Our research indicated that plant-based dietary pattern analyzed by PCA was negatively associated with blood uric acid concentrations, while animal-based dietary pattern was directly associated with blood uric acid concentrations. The RRR dietary pattern may have the potential to induce elevations in blood uric acid concentrations.

**Supplementary Information:**

The online version contains supplementary material available at 10.1186/s12937-022-00789-7.

## Background

Hyperuricemia is a chronic metabolic disease caused by blood uric acid accumulation due to purine metabolism disorders. It is the leading cause of gout and is also related to diabetes, chronic kidney disease, cardiovascular disease, and other diseases [1–4]. Several recent epidemiological surveys have indicated that the prevalence rates of hyperuricemia among adults in the United States, Australia, and South Korea are 20.1%, 16.6%, and 11.4% [5–7], respectively. Moreover, the prevalence of hyperuricemia has increased in recent decades [8]. Therefore, hyperuricemia has become an important public health problem. A growing number of studies have focused on the modifiable risk factors, especially dietary factors [9], of hyperuricemia. In recent years, many studies have investigated the association between individual food or nutrients and hyperuricemia. For example, the risk of hyperuricemia is increased by the excessive intake of alcohol, sugary beverages, and food with high purine contents [10–12] but is negatively correlated with the intake of magnesium and zinc [13, 14]. However, due to the complexity of the diet and the potential interactions between food components, the cumulative effect of various nutrients in the entire diet on hyperuricemia may be different from that of a single nutrient or food [15]. Research on dietary patterns and chronic diseases may give further consideration to the comprehensive effects of food [16]. Many studies have reported on the relationship between dietary patterns and chronic diseases, including hypertension, diabetes and obesity. However, works on the association of dietary patterns with blood uric acid concentration and hyperuricemia remain limited, and their results are inconsistent. For example, one cross-sectional study showed that no significant association exists between the Chinese dietary pattern and blood uric acid concentration [17]. An ATTCI study from Greece indicated that the Mediterranean diet is related to the reduction of blood uric acid concentration [18]. One case–control study demonstrated that the high incidence of newly diagnosed hyperuricemia is associated with the animal food dietary pattern but not with the dietary pattern that is mainly based on the intake of vegetables, bean products, and whole grain cereal [19]. Another cross-sectional study illustrated that the dietary pattern that mainly consists of rice, whole grain cereals, vegetables, soy beans, and bean products is associated with the relatively low risk of hyperuricemia [20]. Given that these studies were limited by small sample sizes or geographical locations, we used the baseline data of one epidemiological study that covered several cities across China to evaluate the association between the dietary patterns of northern Chinese adults and hyperuricemia.

## Methods

### Study population

**Fig. 1:**
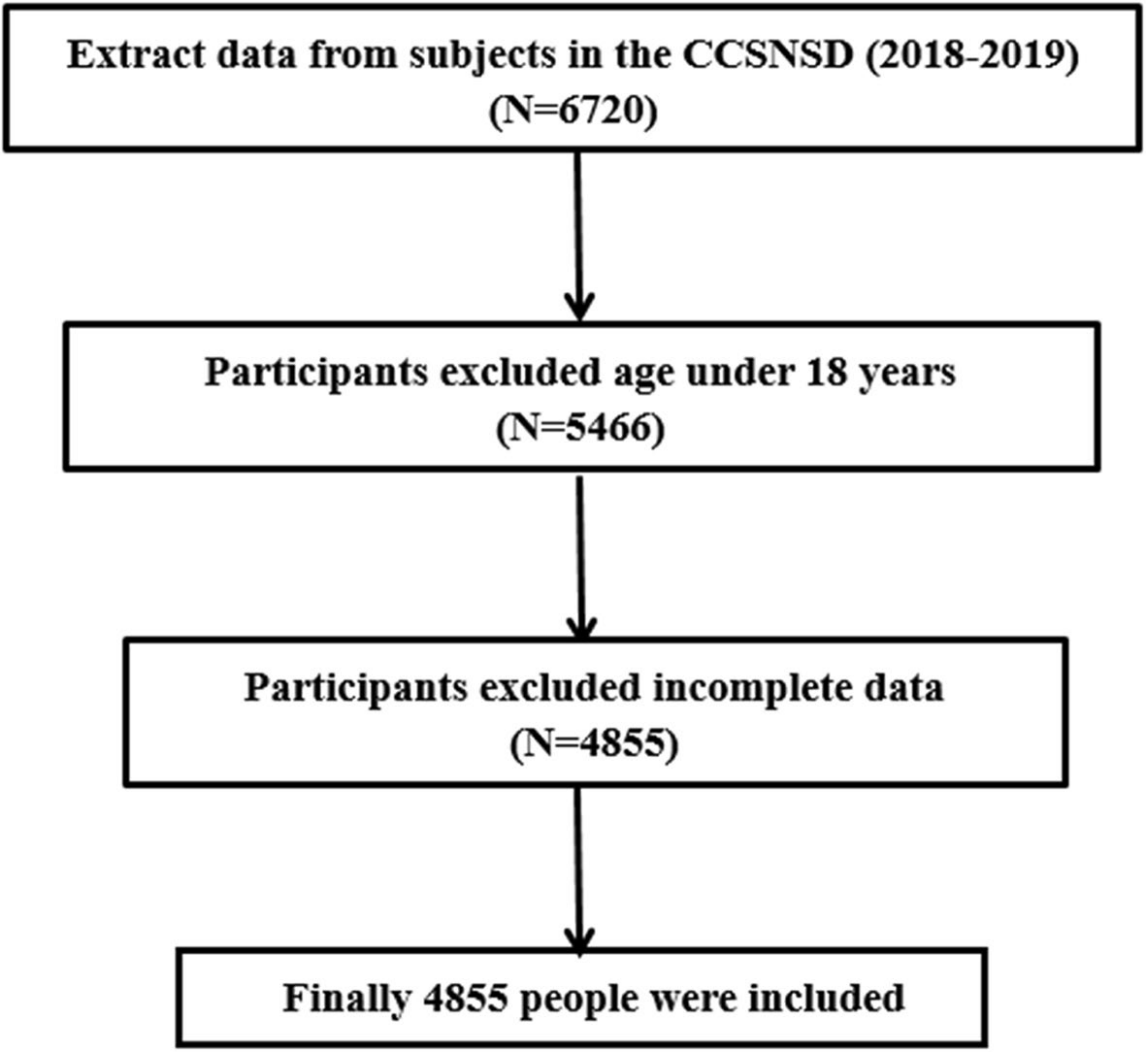
Flow chart of the sampling of the study participants

The data used in this work were from the baseline data of the Community Cohort Study on Specialized Nervous System Diseases (No.2017YFC0907701), which is a National Key R&D Program of China Precision Medicine Project. This study was reviewed and approved by the Institutional Review Board of the National Institute for Nutrition and Health (No. 2017020, 6 November 2017). In this project, the study population was selected in 2018 from four Chinese provinces via the segmented cluster sampling method. Information on the general demographics, disease history, diet, and other lifestyle habits of the research population were collected via face-to-face interviews. Physical examination was performed, and blood samples were collected. In this study, a total of 6720 people, of whom 5466 were adults, from Hebei Province were recruited. A total of 611 people were excluded due to incomplete data. Finally, 4855 people were included (Fig. 1).

### Diet assessment

FFQ was used to collect dietary intake data at the baseline for 65 items. Dietary supplements were excluded. Trained professionals asked the participants about their habitual consumption frequency (daily, weekly, monthly, yearly, or never) of each food item in the reference categories within the past 12 months. Food consumption was converted into daily food intake on a uniform basis and into g/day. In accordance with the Chinese food composition table, the 65 items were classified into 17 categories: cereals and potatoes, legumes and their products, fresh vegetables, mushrooms and algal food, preserved and processed vegetables, fruits, poultry, meat, fish and shrimp, animal organs, processed meats, eggs, snacks and desserts, dairy products, nuts, sugary beverages, and tea or coffee (Table 1).

**Table 1:** Food grouping

Food groups	Food items
Cereals and potatoes	Rice and rice products, flour and flour products, whole grain cereals, coarse cereals, sweet potato, taro
Legumes and their products	Dry soybean, soybean milk, tofu, bean curd skin, green bean, red bean
Fresh vegetables	Leguminous vegetable, stem, leafy and flowering vegetables, cucurbitaceous vegetable, solanaceous vegetables
Fungi and algae	Various mushrooms (fresh), seaweed
Preserved vegetables	Dried vegetables, pickled vegetables, salted vegetables
Fruits	Orange, mandarin, apple, banana, peach, pear, grape, kiwi, watermelon, melon
Poultry	Chicken, duck, goose
Meat	Pork, beef, lamb
Animal organs	Chicken liver, goose liver, duck blood, pig liver
Processed meat	Sausage, pork luncheon meat
Fish and other seafood	Freshwater fish, sea fish, shrimp, crab
Eggs	Chicken egg, duck egg, quail egg
Dairy products	Liquid milk, yogurt, cheese, milk powder
Snacks and dessert	Soda biscuit, cookie, cake, crisps, chocolate
Nuts	Peanut, melon seeds, other nuts
Sugary beverages	Carbonated beverages, juice, sports drinks
Tea and coffee	Green tea, black tea, coffee

### Definitions of other variables

Demographic factors included the following: age in years (18–40, 41–65, and > 65 years); educational level (below junior school, secondary school, undergraduate, and higher). A questionnaire was used to collect the following lifestyle-related information: smoking status (yes or no) and drinking status (yes or no). Current smokers were defined as having smoked at least one cigarette per day. Recent drinking was defined as alcohol consumption at least once in the past 1 year.

Hyperuricemia was defined as serum blood uric acid concentration ≥ 416.5 μmol/L in men and 357 μmol/L in women [21]. BMI was calculated as body weight/height [2] (BMI < 24.0 or BMI ≥ 24.0) [22]. Hypertension was defined as having self-reported hypertension or high blood value systolic blood pressure ≥ 140 mmHg or diastolic blood pressure ≥ 90 mmHg [23]. Diabetes was defined as self-reported diabetes or fasting glucose ≥ 7.0 mmol/L [24]. Dyslipidemia was defined as having more than one of the following conditions: 1) total cholesterol ≥ 6.2 mmol/L; 2) triglyceride ≥ 2.3 mmol/L; 3) low-density lipoprotein cholesterol ≥ 4.1 mmol/L; and 4) high-density lipoprotein cholesterol < 1.0 mmol/L [25].

### Statistical analyses

Data reduction techniques based on PCA, PLS, and RRR were used to identify dietary patterns involving the 17 food groups. The Kaiser–Meyer–Olkin measure of sampling adequacy and Bartlett’s test of sphericity were used to evaluate the adequacy of correlation matrixes with the data. PCA was used to derive the major dietary patterns on the basis of the frequency of the consumption of the 17 food groups in this FFQ. Varimax was applied to maintain factorial independence and increase interpretability. Factors with eigenvalue > 1 were extracted, and then scree plots were used to identify the major dietary patterns. The *PROC PLS* statement in SAS (SAS Institute Inc., Cary, NC, USA) was utilized to conduct RRR and PLS analyses with the definitions “METHOD = PLS” or “METHOD = RRR.” In the analysis, the values of blood uric acid were used as response variables, the 17 food groups were considered as predictors, and one factor was extracted from each of the two methods as the dietary pattern. Food groups with absolute factor loading value > 0.20 were considered to be the main contributors to a dietary pattern and included in this study. A pattern score was obtained for each participant by obtaining the total consumption of each food group weighted by factor loading. A high score indicated that the intake of food groups associated with a specific pattern was high.

The basic characteristics of the participants were described in accordance with the quartile of the scores for each dietary pattern (ascending from Q1 to Q4). The data of continuous variables were expressed as mean ± SD and those of categorical variables were expressed as frequencies (percentage). χ^2^ test and ANOVA were used to compare categorical and continuous variables, respectively. We estimated the associations of dietary pattern factor scores with blood uric acid concentrations by using multivariate linear regression models. Hyperuricemia status was used as the dependent variable for binary logistic regression analysis, and confounders, such as age, gender, smoking, alcohol consumption, blood pressure, and blood lipids, were adjusted. RRR and PLS analyses were performed with SAS version 9.1 (SAS Institute Inc., Cary, NC, USA). All other analyses were conducted by using SPSS software package version 22.0 for Windows (SPSS Inc, Chicago, IL). A two-tailed *P* < 0.05 was considered significant.

## Results

### Characteristics of participants

A total of 4855 participants were included in the present study. The overall prevalence of hyperuricemia was 19.3%. The prevalence of hyperuricemia among males was 22.0% and that among females was 17.3%. The demographic and clinical characteristics of the participants with and without hyperuricemia are shown in Table 2. Significant differences were observed between participants with and without hyperuricemia in terms of age, gender, drinking status, residential zone, obesity, hypertension, and dyslipidemia.

**Table 2:** Baseline characteristics of participants by hyperuricemia status

Character	Total group *n* = 4855	Hyperuricemic		*p*
		NO *n* = 3918 (80.7)	YES *n* = 937 (19.3)	
**Age, n (%)**				< 0.01
18–40 years	1024 (21.1)	766 (74.8)	258 (25.2)	
40–65 years	1879 (38.7)	1529 (81.4)	350 (18.6)	
≥ 65 years	1952 (40.2)	1623 (83.1)	329 (16.9)	
**Gender**, n (%)				< 0.01
Men	2019 (41.6)	1574 (78.0)	445 (22.0)	
Woman	2836 (58.4)	2344 (82.7)	492 (17.3)	
**Residence, n (%)**				< 0.01
Urban	1869 (38.5)	1415 (75.7)	454 (24.3)	
Rural	2986 (61.5)	2503 (83.8)	483 (16.2)	
**Education, n (%)**				
Low	2271 (46.8)	1910 (84.1)	361 (15.9)	< 0.01
Middle	2078 (42.8)	1633 (78.6)	375 (21.4)	
High	506 (10.4)	375 (74.1)	131 (25.9)	
**Current smoker, n (%)**				0.46
Yes	966 (19.9)	778 (80.5)	188 (19.5)	
No	3889 (80.1)	3140 (80.7)	749 (19.3)	
**Recent drink, n (%)**				
Yes	840 (17.3)	623 (74.2)	217 (25.8)	< 0.01
No	4015 (82.7)	3295 (82.1)	720 (17.9)	
**BMI ≥ 24.0, n (%)**				< 0.01
Yes	2834 (58.4)	2131 (75.2)	703 (24.8)	
No	2021 (41.6)	1787 (88.4)	234 (11.6)	
**Hypertension, n (%)**				< 0.01
Yes	2561 (52.7)	2008 (78.4)	553 (21.6)	
No	2294 (17.3)	1910 (83.3)	384 (16.7)	
**Diabetes, n (%)**				0.81
Yes	602 (12.4)	488 (81.1)	114 (18.9)	
No	4253 (87.6)	3430 (80.6)	823 (19.4)	
**TC (mmol/L), (SD)**	4.66 ± 0.99	4.62 ± 0.97	4.83 ± 1.08	< 0.01
**HDL-C (mmol/L), (SD)**	1.29 ± 0.36	1.32 ± 0.35	1.17 ± 0.34	< 0.01
**LDL-C (mmol/L), (SD)**	2.84 ± 0.91	2.83 ± 0.88	2.90 ± 1.00	< 0.01
**TG (mmol/L), (SD)**	1.70 ± 1.66	1.58 ± 1.25	2.18 ± 1.86	< 0.01
**Dyslipidemia, n (%)**				< 0.01
Yes	1623 (33.4)	1156 (29.5)	467 (49.8)	
No	3323 (66.6)	2762 (70.5)	470 (50.2)	

### Dietary pattern identification

The Kaiser–Meyer–Olkin index (0.753) and Bartlett’s test (*P* < 0.01) showed that the correlation among variables was sufficiently strong for factor analysis. Three major dietary patterns were determined by using PCA. The first dietary pattern was characterized by the high intake of fresh vegetables, fruits, dairy products, eggs, and legumes and their products. This pattern was designated as the plant-based pattern. The second type featured the high intake of pickled and processed vegetables, processed meat, snacks, and mushrooms and algal food and was denoted as the processed food pattern. The third type was characterized by the high intake of poultry, livestock, fish and shrimp, processed meats and nuts and was considered as the animal dietary pattern. The dietary pattern obtained through RRR was distinguished by the high intake of poultry meat, sugar-sweetened beverages, animal organs, and tea or coffee, and the low intake of snacks and desserts. The dietary pattern obtained from PLS was similar to that obtained from RRR but had a higher intake of fruits and beans (Table 3).

**Table 3:** Factor loadings of food groups in each dietary pattern identified by using PCA, RRR, and PLS

	**Plant-based diet**	**Processed food diet**	**Animal-based diet**	**RRR diet**	**PLS diet**
**Cereals and potatoes**	− 0.08	− 0.00	0.15	0.02	0.02
Legumes and their products	0.55^a^	0.10	0.09	0.20	0.26^a^
Fresh vegetables	0.70^a^	0.13	0.15	0.10	0.24^a^
Mushrooms and algae	0.19	0.63^a^	− 0.02	− 0.00	0.11
Preserved vegetables	0.02	0.57^a^	− 0.13	0.15	0.15
Fruits	0.60^a^	0.28	0.08	0.11	0.24^a^
Poultry	0.21	0.05	0.67^a^	0.53^a^	0.51^a^
Meat	0.18	0.02	0.68^a^	0.08	0.27^a^
Fish and shrimp	0.26	− 0.14	0.31	− 0.06	0.03
Animal organs	0.16	0.30	0.04	0.38^a^	0.40^a^
Processed meats	− 0.03	0.49^a^	0.32	− 0.21^a^	− 0.12
Eggs	0.43^a^	− 0.15	− 0.06	− 0.16	0.01
Snacks and dessert	0.06	0.55^a^	0.07	− 0.30^a^	− 0.06
Dairy products	0.58^a^	0.18	− 0.11	0.09	0.12
Nuts	− 0.06	0.13	0.32	0.16	0.17
Sugary beverages,	− 0.04	0.20	0.145	0.46^a^	0.40^a^
Tea or coffee	0.11	0.11	0.12	0.29	0.28^a^

^a^Represents the major characteristic food group of the dietary pattern

### Participant characteristics and dietary patterns

We identified the characteristics of the participants on the basis of the quartile of their score for each eating pattern (Table 4). Compared with the participants with scores for the plant-based and processed food diet patterns in the lower quartile, those with scores in the higher quartile were more likely to be urban residents, female, less educated, and nonsmokers. Participants with higher scores for the animal-based diet pattern were more likely to be male, city residents, smokers, and alcohol consumers than those with lower scores. Participants who scored higher for the RRR diet pattern were more likely to live in cities, have a lower BMI, and consume alcohol than those with lower scores. Participants who scored higher on the PLS eating patterns were more likely to be urban alcohol consumers than those who scored lower.

**Table 4:** Characteristics of the study participants by quartile (Q) of dietary pattern scores

		Plant-based diet		*p*	Processed food diet		*p*	Animal diet		*p*	RRR diet		*p*	PLS diet		*p*
		Q1	Q4		Q1	Q4		Q1	Q4		Q1	Q4		Q1	Q4	
Age				0.46			< 0.01			< 0.01			< 0.01			< 0.01
18–40 years	1024(21.1)	266(21.9)	253(20.9)		230(18.9)	301(24.8)		177 (14.6)	339 (27.9)		231 (19.0)	300 (24.7)		212 (17.5)	308 (25.4)	
41–65 years	1879(38.7)	480(39.5)	488(40.2)		462(38.1)	492 (40.6)		449 (37.0)	439(36.2)		451 (37.1)	168 (38.6)		440 (36.3)	475 (39.2)	
Over 65 years	1952(40.2)	468(38.6)	472 (38.9)		522 (43.0)	420(34.6)		588(48.4)	435 (35.9)		535 (44.0)	446 (36.7)		559(46.2)	430 (35.4)	
Female	2836(58.4)	659 (57.2)	749 (61.7)	0.03	653(53.8)	744 (61.3)	< 0.01	772(63.6)	649(53.5)	< 0.01	709 (58.3)	684 (56.3)	0.23	699 (57.7)	690 (56.9)	0.06
Live in cities	1869 (38.5)	251(20.7)	693(57.1)	< 0.01	409 (33.7)	618(50.9)	< 0.01	406(33.4)	592(48.8)	< 0.01	252 (20.7)	685 (56.4)	< 0.01	201 (16.6)	705 (58.1)	< 0.01
Educational level													< 0.01			< 0.01
Below junior school	2271(46.8)	685(56.4)	419(34.5)	< 0.01	624(51.4)	485(40.0)	< 0.01	611(50.3)	511 (42.1)	< 0.01	682 (56.0)	422 (34.8)		705 (58.2)	397 (32.7)	
Secondary school	2078(42.8)	467(38.5)	569(46.9)		486(40.0)	533 (43.9)		496(40.9)	520(42.9)		476 (39.1)	568 (46.8)		463 (38.2)	574 (47.3)	
Undergraduate and higher	506(10.4)	62(5.1)	225 (18.5)		104 (8.6)	195 (16.1)		107(8.8)	182(15.0)		59 (4.8)	224 (18.5)		43 (3.6)	242 (20.0)	
Smoke	966(19.9)	240 (19.8)	205 (16.9)	0.01	239(19.7)	244(20.1)	0.99	206(17.0)	278(22.9)	< 0.01	235 (20.8)	232 (19.1)	0.55	253 (20.9)	233 (19.2)	0.23
Drink alcohol	840(17.3)	159 (13.1)	234(19.3)	< 0.01	176(14.5)	256(21.1)	< 0.01	171(14.1)	253 (20.9)	< 0.01	177 (14.5)	198 (22.7)	< 0.01	159 (13.1)	286 (23.6)	< 0.01
BMI > 23.9	2834 (58.4)	493 (24.4)	490 (24.2)	0.35	510 (25.2)	514 (25.4)	0.36	528 (26.1)	484 (23.9)	0.30	558 (27.6)	490 (24.3)	< 0.01	525 (26.0)	508 (25.1)	0.13
Hypertension	2561(52.7)	660 (54.4)	617(50.9)	0.15	595 (51.0)	596(50.9)	0.07	673(55.4)	612(50.5)	0.11	643 (52.8)	613 (50.5)	0.29	677 (55.9)	654 (49.5)	0.01
Diabetes	602(12.4)	166(13.7)	142 (11.7)	0.46	158(13.0)	141 (11.6)	0.479	151(12.4)	152 (12.5)	0.99	150 (12.3)	151 (12.4)	0.87	156 (12.9)	143 (11.8)	0.66
TC	4.66 ± 0.99	4.64 ± 1.02	4.69 ± 1.00	0.11	4.64 ± 0.96	4.68 ± 1.03	0.782	4.67 ± 0.95	4.63 ± 1.03	0.99	4.63 ± 1.00	1.69 ± 0.99	0.32	4.60 ± 0.93	1.68 ± 1.01	0.08
HDL-C	1.29 ± 0.36	1.29 ± 0.36	1.27 ± 0.34	0.123	1.31 ± 0.36	1.30 ± 0.35	0.227	1.30 ± 0.36	1.31 ± 0.35	0.16	1.32 ± 0.36	1.29 ± 0.35	< 0.01	1.31 ± 0.36	1.28 ± 0.35	0.15
LDL-C	2.84 ± 0.91	2.81 ± 0.9	2.87 ± 0.94	0.27	2.85 ± 0.87	2.87 ± 0.95	0.786	2.85 ± 0.87	2.85 ± 0.95	0.99	2.83 ± 0.91	2.85 ± 0.91	0.79	2.79 ± 0.84	2.87 ± 0.94	0.06
TG	1.70 ± 1.66	1.73 ± 2.01	1.67 ± 1.14	0.76	1.66 ± 1.74	1.68 ± 1.61	0.474	1.69 ± 1.69	1.67 ± 1.65	0.84	1.65 ± 1.96	1.76 ± 1.66	0.16	1.67 ± 1.96	1.70 ± 1.66	0.90
Dyslipidemia	1623 (33.4)	392 (24.2)	424 (26.1)	0.57	397 (24.5)	386 (23.8)	0.201	403 (24.8)	385 (23.7)	0.40	374 (23.0)	427 (26.3)	0.09	375 (23.1)	424 (26.1)	0.19

### Blood uric acid concentrations and dietary patterns

The linear regression relationship between the scores for different dietary patterns and blood uric acid concentrations is shown in Table 5. The plant-based diet pattern score was negatively correlated with blood uric acidconcentrations (β =  − 3.225,95%CI =  − 5.548, − 0.092, *P* = 0.007). The score for the animal dietary pattern was directly correlated with blood uric acid levels (β = 3.654,95%CI = 1.391, 5.918, *P* = 0.002). Values were adjusted for sex, age, residence, educational status, alcohol status, smoking status, BMI, hypertension, diabetes, and dyslipidemia.

**Table 5:** Dietary pattern scores from multiple statistical methods and blood uric acid levels

	Β value	95% CI	*P*
Plant-based diet	− 3.22	(− 5.52, − 0.10)	0.01
Processed food diet	1.12	(− 1.04, 3.44)	0.31
Animal diet	3.65	(1.39, 5.92)	< 0.01
RRR diet	1.62	(− 0.54, 3.78)	0.14
PLS diet	0.61	(− 1.19, 2.41)	0.41

Models were adjusted for sex, age, residence, educational status, alcohol status, smoking status, BMI, hypertension, diabetes, and dyslipidemia

In logistic regression analysis, the highest quartile of plant-based dietary pattern scores was associated with a low risk of hyperuricemia (OR = 0.699; 95% CI: 0.561–0.870,* P* < 0.05), whereas the highest quartile of animal diet pattern scores was associated with a high risk of hyperuricemia (OR = 1.401; 95% CI:1.129–1.739, *P* < 0.05). For the RRR dietary pattern, the ratio of Q3 to Q1 was statistically significant (OR = 1.421;95% CI: 1.146–1.763,* P* < 0.05) (Table 6).

**Table 6:** The ORs (95% CIs) for the association between the quartiles of dietary pattern scores and hyperuricemia

	Quartiles of dietary patterns scores				*P*
	Q1	Q2	Q3	Q4	
Plant-based					
Model 1	1.00	0.85 (0.69–1.04)	0.90 (0.74–1.10)	0.88 (0.72–1.08)	0.42
Model 2	1.00	0.80 (0.65–0.98)*	0.81 (0.66–0.99)*	0.74 (0.60–0.91)*	0.03
Model 3	1.00	0.78 (0.63–0.96)*	0.812 (0.68–1.00)*	0.70 (0.56–0.87)*	0.01
Processed food diet					
Model 1	1.00	1.00 (0.81–1.23)	1.025 (0.83–1.26)	1.25 (1.02–1.52)*	0.08
Model 2	1.00	1.01 (0.82–1.24)	1.002 (0.81–1.23)	1.18 (0.97–1.450)	0.27
Model 3	1.00	0.99 (0.80–1.23)	0.94 (0.76–1.16)	1.19 (0.96–1.46)	0.12
Animal diet					
Model 1	1.00	1.38 (1.12–1.70)*	1.18 (0.95–1.46)	1.42 (1.16–1.76)*	< 0.01
Model 2	1.00	1.41 (1.14–1.74)*	1.20 (0.97–1.48)	1.40 (1.14–1.73)*	< 0.01
Model 3	1.00	1.38 (1.11–1.71)*	1.180 (0.95–1.47)	1.40 (1.13–1.74)*	< 0.01
RRR diet					
Model 1	1.00	1.32 (1.07–1.63)*	1.60 (1.30–1.97)*	1.38 (1.11–1.70)*	< 0.01
Model 2	1.00	1.30 (1.05–1.61)*	1.53 (1.24–1.89)*	1.24 (0.10–1.54)	< 0.01
Model 3	1.00	1.25 (1.00–1.56)*	1.42 (1.15–1.76)*	1.14 (0.91–1.42)	0.01
PLS diet					
Model 1	1.00	1.08 (0.87–1.33)	1.23 (0.10–1.50)	1.20 (0.98–1.48)	0.18
Model 2	1.00	1.05 (0.85–1.29)	1.13 (0.91–1.39)	1.04 (0.84–1.29)	0.73
Model 3	1.00	1.02 (0.83–1.27)	1.07 (0.86–1.32)	0.99 (0.79–1.23)	0.90

Model 1 was adjusted for gender and age

Model 2 was additionally adjusted for residence, education level, alcohol consumption, and smoking status

Model 3 was additionally adjusted for BMI, hypertension, diabetes, and dyslipidemia

^*^P < 0.05

## Discussion

In the present study, the relationship between Chinese dietary patterns and serum uric acid was investigated by using three statistical methods, namely, PCA, RRR and PLS. The plant-based dietary pattern determined by PCA was negatively correlated with serum uric acid, whereas the animal-based dietary pattern was directly correlated with serum uric acid. Although no significant association was found between RRR and PLS dietary patterns and serum uric acid concentrations, the RRR dietary pattern may be a potential risk factor for hyperuricemia.

Similar to the Mediterranean and DASH diets, the plant food dietary pattern is characterized by the intake of large quantities of fresh vegetables, fruits, beans, dairy products, and eggs. Two studies have shown that the Mediterranean and DASH diets are associated with the low risk of hyperuricemia [26, 27]. Vegetables and fruits contain large amounts of vitamins, and studies have demonstrated that vitamin C, folate acid, vitamin B_6_, and vitamin B_12_ may have important functions in reducing blood uric acid concentrations. Vitamin C may regulate blood uric acid concentration via its uricosuric effect. Increasing the concentration of vitamin C may competitively inhibit the reabsorption of uric acid given that vitamin C and uric acid are reabsorbed through anion exchange transport in proximal tubules [28]. Folate acid may reduce the production of uric acid by inactivating xanthine oxidoreductase, which is the key enzyme in the oxidation of hypoxanthine into xanthine [29]. Studies have demonstrated that the intake of dietary fiber is negatively associated with the risk of hyperuricemia. Dietary fiber may bind to uric acid in the intestinal tract and promote the excretion of uric acid [30]. Soy bean isoflavones may reduce the production of uric acid by inactivating the enzymes that oxidize hypoxanthine into xanthine [31].

Consistent with previous studies, this work found that the animal food dietary pattern is directly associated with uric acid concentration and hyperuricemia risk. This association can be ascribed to the following aspects: First, considering that animal foods are normally high in purine, the high intake and accumulation of purine increase uric acid [19]. At same time, the high energy content of animal foods further leads to the increased risk of obesity, which has a strong correlation with hyperuricemia [11]. In addition, animal foods are often rich in proinflammatory nutrients. One recent research showed that a high proinflammatory diet score is associated with the relatively high risk of hyperuricemia. The high production of uric acid or the unusual excretion of uric acid may further lead to hyperuricemia under inflammatory conditions [32]. Furthermore, in the present study, we found that the incidence of hyperuricemia was higher in young adults than in the elderly likely because young adults have higher scores for the animal food dietary pattern. We hypothesized that dietary factors may be an important reason for this difference compared to previous studies.

The processed food diet pattern is not mentioned in previous reports. With the development of the economy and the increase in social pressure, the number of people who may prefer the processed food dietary pattern is increasing because processed foods are convenient to eat and have long storage times. This situation may encourage the development of a new dietary pattern. In the present study, we found no significant association between the processed food diet pattern and hyperuricemia. However, previous studies have reported that the consumption of processed food is associated with the increased risk of cardiovascular disease [33, 34]. The inconsistency between our results and previous results may be attributed to the low processing level of the processed foods in the present study. Given that processing levels may affect chronic diseases differently, foods with low processing levels may have no effect on certain chronic diseases [35].

The potential interactions between different food ingredients remain worthy of in-depth study. Although our results did not reveal significant associations between serum uric acid levels and RRR dietary patterns, we speculated that the low intake of snacks and desserts in the RRR dietary pattern may offset the effect of meat on serum uric acid concentrations. After adjustment for confounding factors, we found that for the RRR dietary pattern, scores in the third quartile were directly correlated with hyperuricemia compared to scores in the first quartile. Therefore, we speculated that the RRR dietary pattern may have a potential positive correlation with blood uric acid concentration and hyperuricemia. Although the PLS and RRR dietary patterns have high intakes of animal foods, such as animal organs and poultry, vegetable and bean intake was higher in the PLS dietary pattern than in the RRR dietary pattern. The complex nature of this pattern may explain this finding to some extent. Although the high intake of animal food increases blood uric acid concentration, the intake of large quantities of vegetables and fruits accelerates the metabolism of blood uric acid. The potential interactions between different foods and nutrients have promoted these results.

To summarize, we discussed the relationship between several dietary patterns and blood uric acid concentration. Although blood uric acid may induce oxidative stress, atherosclerosis, vascular inflammation, and other tissue damage, it also has protective roles by acting as an antioxidant that scavenges free radicals and reducing the permeability of the blood–brain barrier [36–39]. We should control uric acid levels within a reasonable range instead of reducing them blindly. Uric acid mainly originates from the metabolites and derivatives of ATP in the body and from purines in food. Controlling the source and amount of purine intake from food will become a very important problem.

The present study has some limitations. First of all, it is a cross-sectional survey. Therefore, the judgment of causality is limited. Further prospective longitudinal studies are needed to prove causality. Second, we obtained the data on dietary intakes on the basis of the memory of the participants. Such data may have some deviation from the actual intake. Finally, this study did not include the intake of dietary supplements by the participants.

## Conclusions

The plant-based dietary pattern established by PCA was negatively correlated with serum uric acid concentrations, whereas the animal dietary pattern was directly correlated with serum uric acid concentrations. Although the RRR dietary pattern did not show a linear relationship with uric acid concentrations, it does have the potential for inducing increased serum uric acid concentrations. The PLS dietary pattern was more balanced than other patterns and had no relationship with uric acid concentrations. Further longitudinal studies are needed, and dietary uric acid must be maintained within a reasonable range.

## Supplementary Information

**Additional file 1**. Sensitivity analysis. 

## Data Availability

The datasets generated and analysed during the current study are not publicly available due to the research is still in progress.

## Abbreviations

PCA: Principal component analysis
RRR: Reduced-rank regression
PLS: Partial least-squares
FFQ: Food frequency questionnaire
BMI: Body mass index
TC: Total cholesterol
TG: Triglyceride
LDL-C: Low-density lipoprotein cholesterol
HDL-C: High-density lipoprotein cholesterol
