# Supplementary material for: Association of dietary patterns with blood uric acid concentration and hyperuricemia in northern Chinese adults
Source: Nutr J. 2022 Jun 23;21:42. doi: 10.1186/s12937-022-00789-7 (PMC9219223; doi:10.1186/s12937-022-00789-7)
Supplement: Supplementary file 1 — Additional file 1. Sensitivity analysis. [file 12937_2022_789_MOESM1_ESM.docx]

Additional file 1

**Sensitivity analysis:**

Associations between dietary patterns and blood uric acid concentration were evaluated via sensitivity analysis. When evaluating relationships between one certain dietary pattern and blood uric acid concentration, we adjusted other dietary patterns on the basis of the original model.

Table S1 Sensitivity Analysis

|  | Β value | 95% CI | *P* |
| --- | --- | --- | --- |
| **Plant-based diet** | −3.56 | (−0.68,−0.34) | 0.03 |
| **Processed food diet** | 1.12 | (−1.97, 2.81) | 0.73 |
| **Animal diet** | 2.17 | (−0.48, 4.83) | 0.11 |
| **RRR diet** | 2.67 | (−1.27, 6.61) | 0.19 |
| **PLS diet** | −1.73 | (−4.38, 0.91) | 0.20 |

Models were adjusted for sex, age, residence, educational status, alcohol status, smoking status, BMI, hypertension, diabetes, dyslipidemia, and other dietary pattern scores
